# A study of clinical and information management processes in the surgical pre-assessment clinic

**DOI:** 10.1186/1472-6947-14-22

**Published:** 2014-03-25

**Authors:** Matt-Mouley Bouamrane, Frances S Mair

**Affiliations:** 1 Institute of Applied Health Sciences, University of Aberdeen, Scotland, UK; 2 College of Medical, Veterinary and Life Sciences, Institute of Health & Well-Being, University of Glasgow, Scotland, UK

**Keywords:** *(Mesh)*, Perioperative nursing, Medical informatics applications, Information systems

## Abstract

**Background:**

Establishing day-case surgery as the preferred hospital admission route for all eligible patients requires adequate preoperative assessment of patients in order to quickly distinguish those who will require minimum assessment and are suitable for day-case admission from those who will require more extensive management and will need to be admitted as inpatients.

**Methods:**

As part of a study to elucidate clinical and information management processes within the patient surgical pathway in NHS Scotland, we conducted a total of 10 in-depth semi-structured interviews during 4 visits to the Dumfries & Galloway Royal Infirmary surgical pre-assessment clinic. We modelled clinical processes using process-mapping techniques and analysed interview data using qualitative methods. We used Normalisation Process Theory as a conceptual framework to interpret the factors which were identified as facilitating or hindering information elucidation tasks and communication within the multi-disciplinary team.

**Results:**

The pre-assessment clinic of Dumfries & Galloway Royal Infirmary was opened in 2008 in response to clinical and workflow issues which had been identified with former patient management practices in the surgical pathway. The preoperative clinic now operates under well established processes and protocols. The use of a computerised system for managing preoperative documentation substantially transformed clinical practices and facilitates communication and information-sharing among the multi-disciplinary team.

**Conclusion:**

Successful deployment and normalisation of innovative clinical and information management processes was possible because both local and national strategic priorities were synergistic and the system was developed collaboratively by the POA staff and the health-board IT team, resulting in a highly contextualised operationalisation of clinical and information management processes. Further concerted efforts from a range of stakeholders are required to fully integrate preoperative assessment within the health-board surgical care pathway. A substantial – yet unfulfilled – potential benefit in embedding information technology in routine use within the preoperative clinic would be to improve the reporting of surgical outcomes.

## Background

The *'High Impact Changes for Service Improvement and Delivery’* report recommended establishing day-case surgery as the preferred hospital admission route for all eligible patients in order to make more efficient use of hospital resources [1]. A recent report by the National Confidential Enquiry into Patient Outcome and Death (NCEPOD) in the U.K. found that 16% of the hospitals reviewed had no pre-admission anaesthetic assessment clinic, 17% had no surgical assessment clinic and that nearly 20% of elective high-risk patients were not seen in a preoperative clinic prior to surgery [2]. Patients undergoing surgery are increasingly older, often have complex chronic morbidities and require careful preoperative planning. The NCEPOD report emphasised the importance of high-quality preoperative assessment (POA) to ensure the early identification and effective clinical management of 'higher-risk’ patients, in order to reduce surgical mortality rates. To establish day-case surgery as the preferred hospital admission route for all *eligible* patients therefore requires the prior assessment of *all* patients in order to quickly distinguish those who are suitable for day-case admission from those who will require more extensive management and will need to be admitted as inpatients. The NHS Modernisation Agency Preoperative Assessment Project (2001-2002) estimated that up to two-thirds of day-case cancellations and half of inpatient cancellations were directly due to patient-related factors and that more effective POA processes could significantly reduce cancellation rates [3,4]. Effective patient evaluation will depend upon the efficient collection of all appropriate medical information, good data management and communication between the members of the multi-disciplinary team (MDT) and the adequate use of actionable knowledge to decide on an appropriate course of action for optimum patient management [5,6].

There is an overall lack of robust evidence in the literature about how preoperative services should be organised in practice [7]. A recent literature review of post-operative recovery following day-case surgery has highlighted a lack of evidence on which to base innovative nursing care and education and emphasised the importance of promoting a co-ordinated approach to the nurse-patient communication and information provision throughout the patient surgical pathway [8]. Several recent studies have emphasised the benefits of preoperative assessment clinics, including: identifying undiagnosed medical problems, improving the management of operating room resources, reducing surgical cancellations and delays and improving patient satisfaction [9,11]. While much has been reported on clinical processes in preoperative assessment – on the other-hand – the literature on organisational aspects of preoperative clinics in the NHS remains scarce and more research is necessary to understand how these services operate in order to identify areas of best practice [12,13].

As part of a study on information management processes in the patient surgical pathway, we visited preoperative clinics in all 14 territorial health-boards of NHS Scotland, as well as conducting semi-structured interviews with primary care practitioners between February 2011 and January 2013 [6,14-16]. Day surgery pathways and surgical pre-assessment clinics (PAC) have only been developed relatively recently in NHS Scotland: most were developed in the last 10 to 15 years; several of them only in the last 5 years – as is the case of the preoperative clinic described in this study. At the time of our initial visits, all but two of the NHS health boards (Greater Glasgow and Clyde, GGC and Dumfries and Galloway) provided preoperative services relying on paper-based clinical records and processes. We have highlighted in other studies how the use of electronic vs. paper patient records has specific implications for information access and sharing across the patient care pathway [14-16].

New technologies often fail to 'normalise’ because the disruption caused to professional relationships and ways of working can lead to the rejection of new systems [17-19]. In that respect, the PAC development in Dumfries and Galloway Royal Infirmary (DGRI) presents several unique aspects in comparison to other sites in Scotland. The clinic was developed as part of the Planned Care Improvement Programme (PCIP) and the deployment of an electronic preoperative information system was an intrinsic and central element of the design of the new clinic [20]. Key aspects of the PAC development include: the design of a patient-centred clinical pathway adapted to the health-board local context and priorities and a purposely developed electronic preoperative information management system, which facilitates communication and information-sharing among the various members of the multi-disciplinary team (MDT). The software specifications were designed iteratively by the preoperative staff in collaboration with the local NHS health-board Information Technology (IT) team and the new electronic information system was 'organically’ deployed as part of the new PAC.

## Methods

### Data collection

Ethical approval for this study was obtained in February 2011 from the University of Glasgow College of Medicine ethics committee. The results presented in this study relate specifically to 4 visits to DGRI: an initial visit took place in April 2011 with 3 follow-up visits in April 2012, November 2012 and January 2013. We conducted 10 face-to-face interviews with 5 participants: 3 interviews (baseline & follow-up) with a preoperative nurse (Nurse 1), 4 interviews (baseline & follow-up) with an anaesthetist, 1 joint interview with 2 auxiliary nurses (baseline, nurse 2 & 3) and 2 interviews with an IT support staff (baseline & follow-up, IT 1). Seven interviews were recorded with the explicit consent of each individual respondent and transcribed verbatim, with a mean duration of approximately 50 minutes per interview. A further three interviews were conducted using field notes: one with IT 1 in April 2011 and a further two with Nurse 1 and Anaesthetist 1 in November 2012.

The interviews aimed to collect respondents’ views around the following core themes: *interviewee’s background and training, overview of the preoperative service, key steps within the patient preoperative pathway, patient medical history collection, screening and risk assessment, the use of protocols and guidelines, roles and responsibilities within the multi-disciplinary team, parallel processes (i.e. referrals to other services) and information management and usage processes*. The interviews were semi-structured and open-ended in order to allow the interviewer or interviewee to elaborate on unanticipated and potentially valuable information with additional questions, and probe for further explanation [21]. In addition, we collected all relevant POA documentation which was provided to us by the PAC staff, such as printed copies of the patient integrated care pathway, all documented risk assessment protocols and guidelines as well as relevant documentation for preoperative screening and referrals to other specialist services.

### Data analysis

We analysed data collected using process-mapping techniques and qualitative data analysis [22]. A process map is a visual representation model of a set of clinical services [23-25]. The aim of process mapping is to provide the agents involved in the services, or those analysing or planning an intervention within these services, with detailed models depicting existing care pathways and clinical processes in order to provide an overall picture of how the services are currently provided and how they are performing.

With the increased recognition that complex interventions or innovative technologies can be difficult to implement in practice or often fail to become embedded into routine work practices, there has recently been a growing interest in using theories to provide the foundation for analysing and understanding the socio-technical factors which can explain why complex interventions can succeed or fail in a given context [26-28]. We used Normalisation Process Theory (NPT) as a conceptual framework to interpret the factors which were identified as facilitating or hindering information elucidation tasks and communication within the preoperative multi-disciplinary team.

NPT is concerned with the social organisation of the work *(implementation)* of making practices routine elements of everyday life *(embedding)* and of sustaining embedded practices in their social contexts *(integration)* and was developed particularly in response to the evidence, which suggested that embedding and integrating innovative health interventions can be difficult to achieve in practice [29-31]. NPT was chosen as the overarching theoretical framework for the interpretation of the results of this study (and related work) as it considers a complex intervention – such as the introduction of new services or a technology implementation – as a dynamic social process, shaped by the collective action of stakeholders through their 'agency’, i.e. the ability for health professionals to shape events on the ground through one’s own actions. In addition, NPT considers the organisational context in which the stakeholders’ agency operates as being a key factor affecting the ultimate normalisation – or not – of new services and systems. In the context of the relatively recent (mostly within the last 5 to 10 years) development of PACs across NHS Scotland, national strategic priorities have proved to be crucial in the development and implementation of integrated preoperative services. NPT thus provides a useful theoretical lens through which one can understand how new systems and services (such as electronic health records and integrated care pathways) can be successfully deployed, what are the factors that contribute to the normalisation of new ways of working or – on the other hand – what were the factors which may have contributed to the failure or rejection of new systems and services.

## Results

### Rationale for the development of a pre-operative assessment clinic

The PAC at DGRI started operating in July 2008, in order to cover pre-assessment for general surgery and other surgical specialties. Before then, pre-assessment was already routinely performed for patients scheduled for day-surgery, orthopaedic patients and in the children’s ward. However, for general surgery, patients would traditionally present as inpatients the night before surgery without prior anaesthetic assessment. This could lead to surgery having to be cancelled at short notice if medical issues were identified following admission. In some cases, the surgical procedure would proceed as planned, albeit with a patient whose fitness was not necessarily optimum for surgery. The anaesthetist interviewed suggested that this situation was clearly deemed unsatisfactory by various stakeholders within the hospital. A dedicated PAC could avoid many cases of late surgical cancellation. Non-optimal presentation for surgery could also be remedied if clinical problems with the patients were identified during pre-assessment a few weeks prior to the planned surgical procedure.

**∙ Anaesthetist 1:***“[...] it was really born out of frustration with the previous system whereby, the traditional approach would be that a patient would turn up the night before surgery... no-one having seen him before, or any anaesthetic input, and then often you would discover problems that – if you’d seen them some weeks before – could have been remedied. So it was leading to frustrations both professionally and for the patient being cancelled at short notice... and I was well aware that these systems were up and running in many of the other parts of the country and other parts of the world”.*

In addition, all NHS health-boards in Scotland are now under statutory obligations to ensure that patients are treated within 18 weeks of an initial referral [32]. A surgical procedure cancelled at the last minute would naturally mean that meeting the target would be difficult – if not impossible – for those patients affected. Furthermore, the ratio of day-case surgery and outpatient procedures vs. inpatient procedures was also set as a key strategic health policy priority under the NHS Health, Efficiency, Access and Treatment (HEAT) programme. The HEAT target tasked all health-boards to achieve a minimum of 80% of British Association of Day Surgery (BADS) surgical procedures performed as either day-case or outpatient procedures by March 2011 [33,34].

With the support of the hospital management, the process of creating a dedicated PAC was initiated and a multi-disciplinary working group was established in 2007. The working group included a member of the hospital management, the patients’ manager, anaesthetists, surgeons, an occupational therapist, a physiotherapist, district nurses and one General Practitioner (GP). A key motivation behind the PAC development was therefore to optimise patients’ preparation for surgery and improve the management of hospital resources. It was anticipated that the PAC would cover pre-assessment of all adult patients scheduled for elective surgery. Furthermore, an additional aim of the PAC was to reduce the ratio of patients admitted as inpatients and ensure that patients who were triaged at the PAC as being of 'low-risk’ of perioperative complications could be admitted for day-case surgery or 23-hours stay, thus reducing the demand on beds and associated hospital resources.

### PAC service model

**Figure 1:**
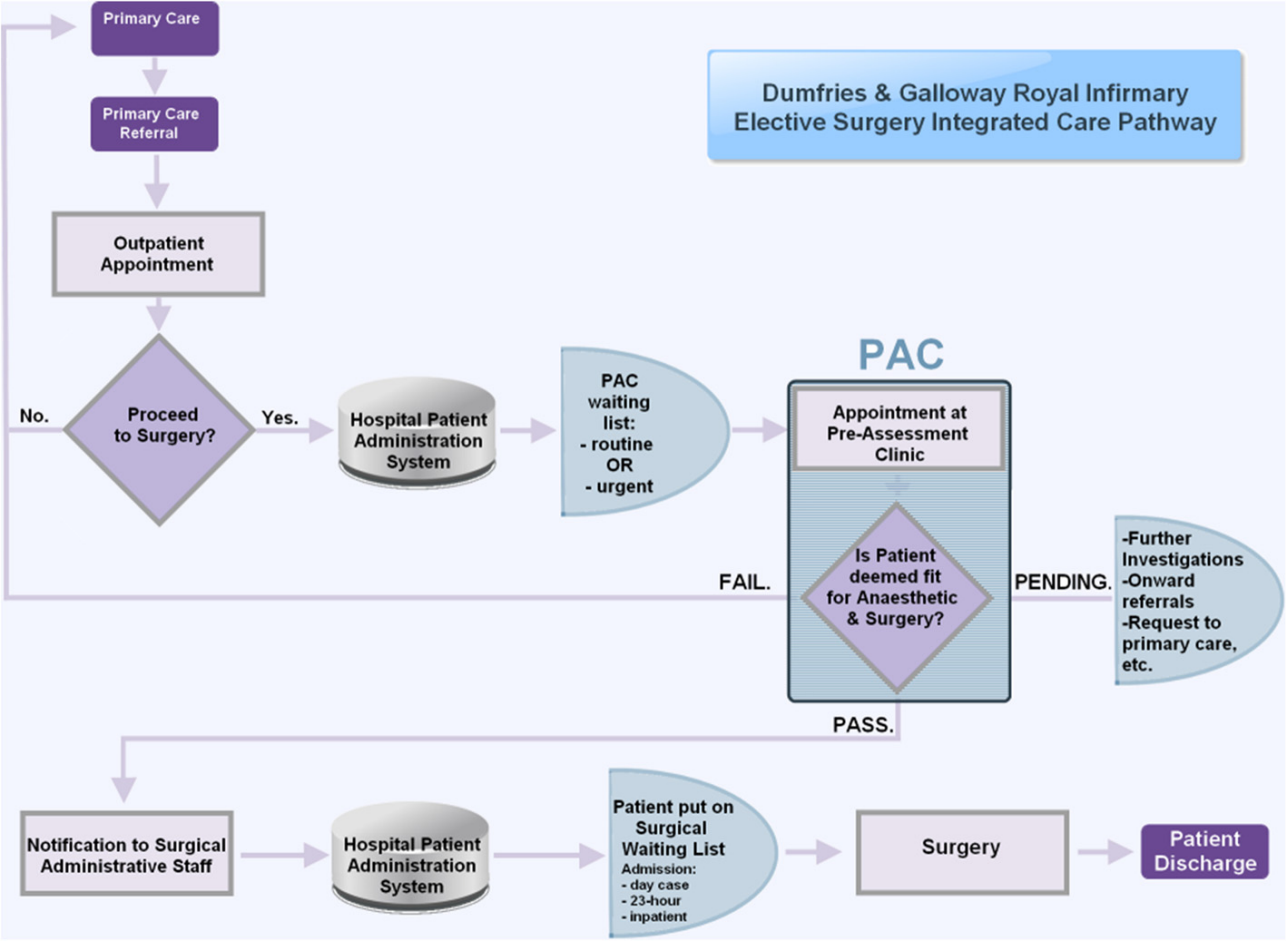
Overview of the Integrated Care Pathway for elective surgery (Dumfries & Galloway Royal Infirmary - Feb. 2013).

Following referral from his GP, a patient will be seen by a specialist consultant (surgeon) at the outpatient clinic. If surgery is deemed necessary by the consultant, the patient will then be referred to the PAC and put on a waiting list (see Figure 1). The PAC initially operated a *“one-stop clinic”* service model: a patient would immediately present at the PAC following on from his appointment with the surgical consultant at the outpatient clinic. However, this model introduced workflow problems as the clinic would initially be very quiet early on in the morning. Later in the day, there would be sudden influxes of patients and the nurses could not manage to see them all immediately, leading to significant patient queues before assessment. Long waiting times impact negatively on patients’ experiences and satisfaction in the perioperative pathway [35]. In order, to avoid patients having to wait for an appointment, the initial service model was then replaced with a booking system. Three slots a day are reserved for urgent referrals such as cancer patients, or patients needing surgery within a week. Those can be referred immediately from the outpatient clinic and a nurse will interview them at the earliest opportunity. For other non-urgent elective patients, the PAC aims to ensure that patients are seen within a few weeks of the consultant having made the decision to operate on the patient, in keeping with the 18 weeks Referral-To-Treatment (RTT) target.

### Training, roles and responsibilities in the PAC

### Competency training in surgical preoperative assessment

The PAC is managed by a senior preoperative nurse who had extensive experience of day-surgery before taking on the responsibility of leading the development of the new PAC. The PAC was designed over 12 months and incorporated the nursing staff responsible for pre-assessment within the day-surgery unit. All the nurses who joined the new clinic also received in-house training in POA. Two competency framework documents were designed by the nurse in consultation with the anaesthetists: one for registered nurse and one for auxiliary nurse staff. The nurses also liaised closely with the anaesthetists for POA training.

**∙ Nurse 1:***“[...] I had done pre-assessment for many years. One of the nurses that came on board when we opened the unit, came from day-surgery, so she was very experienced. What I did in the 6 months before we opened the unit, was that other nurses that we employed got them... we took over day-surgery pre-assessment and got the nurses, the other nurses trained in the pre-assessment process for day-surgery and then we liaised closely with the anaesthetists for training issues [...] Anaesthetists gave them some training on airway assessment”*

Anaesthetist 1 commented that there was not – at the time of the interviews – any structure in place for formal continuing professional development and education for the nursing staff. However, much of the knowledge update and transfer among nurses and between nurses and anaesthetists took place informally during 3 regular weekly anaesthetist-led clinics at the PAC, which we will later describe in more details.

### Roles and responsibilities within the POA multi-disciplinary team

The study of health professionals’ beliefs and behaviours is essential to understand how care pathway re-design can provide effective interventions [36,37]. As the PAC integrated clinical staff from other services, it is essential that each member of the MDT has a clear understanding of his role and responsibilities within the team, as well as knowing when to seek further clinical advice when necessary [38]. In addition to the senior nurse, the PAC is staffed by 4 registered nurses, 3 auxiliary nurses and 2 receptionists who perform administrative duties. A consultant anaesthetist acts as the clinical lead for the service.

#### 

*Role of the auxiliary nurse.* The POA patient interviews begins with the auxiliary nurses asking patients to confirm their demographic details and hospital number and taking the patients for base-line observations. All information, observations collected and tests performed are thoroughly documented and entered into a dedicated electronic preoperative integrated care pathway (ICP) which was developed by the NHS Dumfries & Galloway IT staff in cooperation with the PAC staff. This initial encounter lasts approximately 5 minutes and the patient is then handed over to a nurse for a formal pre-assessment interview. Once the interview is completed, the nurse may request further tests at which point the patient is handed back over into the care of the auxiliary nurse. The auxiliary nurses do not have access to the laboratory test results and these are assessed by the relevant nurse in the computer laboratory system as they become available.

#### 

*Role of the POA nurse.* The nurse sees the patient for a comprehensive structured pre-assessment interview and may choose to carry out any appropriate diagnostic tests. Routine screening tests are usually carried out in the clinic. The nurses also decide whether they need to discuss specific patient cases with an anaesthetist or even re-book an appointment for potentially 'higher-risk’ patients to come back for a full anaesthetic assessment.

Once the nurses receive the screening test results the patient may be deemed suitable for surgery if there are no further concerns (Figure 1). The nurses then inform either the waiting-list team or the relevant surgical secretary that the patient is ready to proceed to surgery. If any of the tests return abnormal results, the nurses would discuss the case with one of the consultants during one of the dedicated anaesthetic clinics. Occasionally, the nurses will also contact the patient’s GP and may ask for a medical intervention, such as administering a treatment for specific conditions (e.g. an infection or undiagnosed hypertension).

**∙ Nurse 1:***“It’s a nurse-led service with anaesthetic lead. We have got a consultant anaesthetist in the department 3 times a week running clinics, so that the nurses initially see the patients, carry out an assessment and if we feel that a patient needs to come back to see a consultant anaesthetist we can bring them back”.*

**Interviewer:** “[...] What’s the protocol for deciding whether... patients should see the anaesthetist?"

**Nurse 1:***“...it depends, a few issues really: nurses’ discretion... Patient arrives at pre-assessment and, you know, they’ve got several co-morbidities and the nurse thinks they might be an anaesthetic risk associated with surgery, they would bring the patient back. Sometimes the consultant surgeons, em... in advance ask us to get patients seen specifically by a consultant anaesthetist. And patients for major vascular surgery, we usually always book those in [...] I think being an anaesthetic department and having clinicians who are, em... you know a lead clinician being an anaesthetist and having an anaesthetist in the department makes a huge difference. I have spoken to other pre-assessment nurses* (i.e. in other hospitals) *who have to go hunting for an anaesthetist if there are issues”.*

#### 

*Role of the Anaesthetist.* The POA service includes 3 dedicated anaesthetic clinics a week, lasting half-a-day. The clinics take place in the afternoon so that some of the more 'complex’ patients assessed in the morning can be asked to return later on that day if it is feasible for them to do so. If a patient can not attend an appointment in the afternoon, then the administrative staff will book a follow-up appointment, usually within one to two weeks. Each anaesthetic clinic is attended by one consultant anaesthetist on a rota and the clinical lead attends the clinic one afternoon a week. Up to five anaesthetists are usually involved in the rota. These clinics will involve the anaesthetists either reviewing the case-notes of the more complex patients or conducting a formal assessment of the patient, including a physical examination, usually to identify potential heart or respiratory issues for anaesthesia or surgery.

**∙ Anaesthetist 1:***“[...] there are 3 sessions* (a week) *in which anaesthetic consultants attend [...] And they’re for patients who either have a direct referral from the surgeon saying 'that I specifically want this patient to be seen by a consultant anaesthetist’ or the... by the nurse they’re... patients who’ve been filtered by the nurse who appreciate there’s a problem, so they send them to us”.*

### The DGRI preoperative care integrated pathway

**Figure 2:**
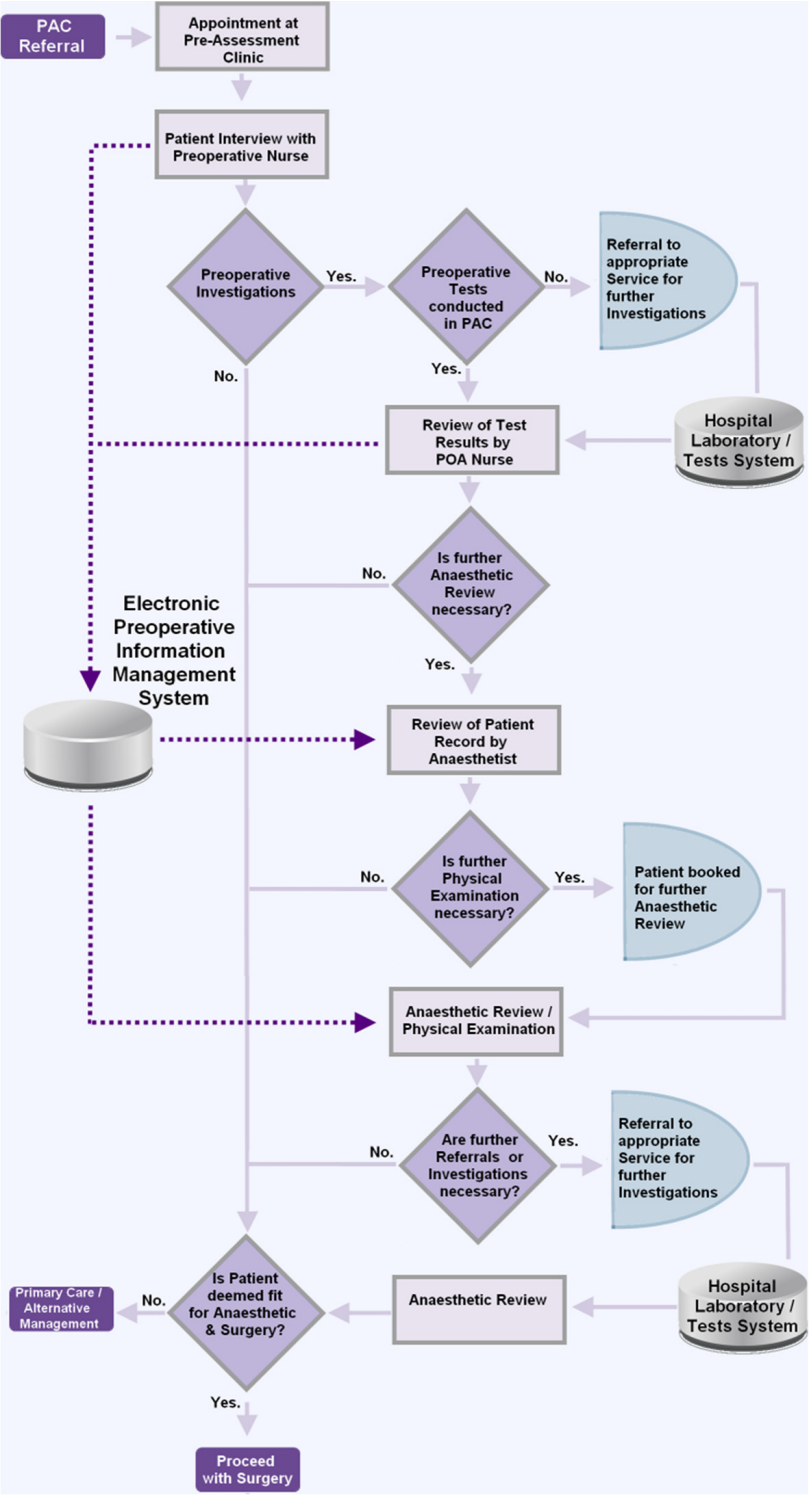
Integrated Care Pathway for Preoperative Assessment (Dumfries & Galloway Royal Infirmary - Feb. 2013).

We modelled the patient preoperative pathway in a process map illustrated in Figure 2.

### The patient preoperative interview

**Figure 3:**
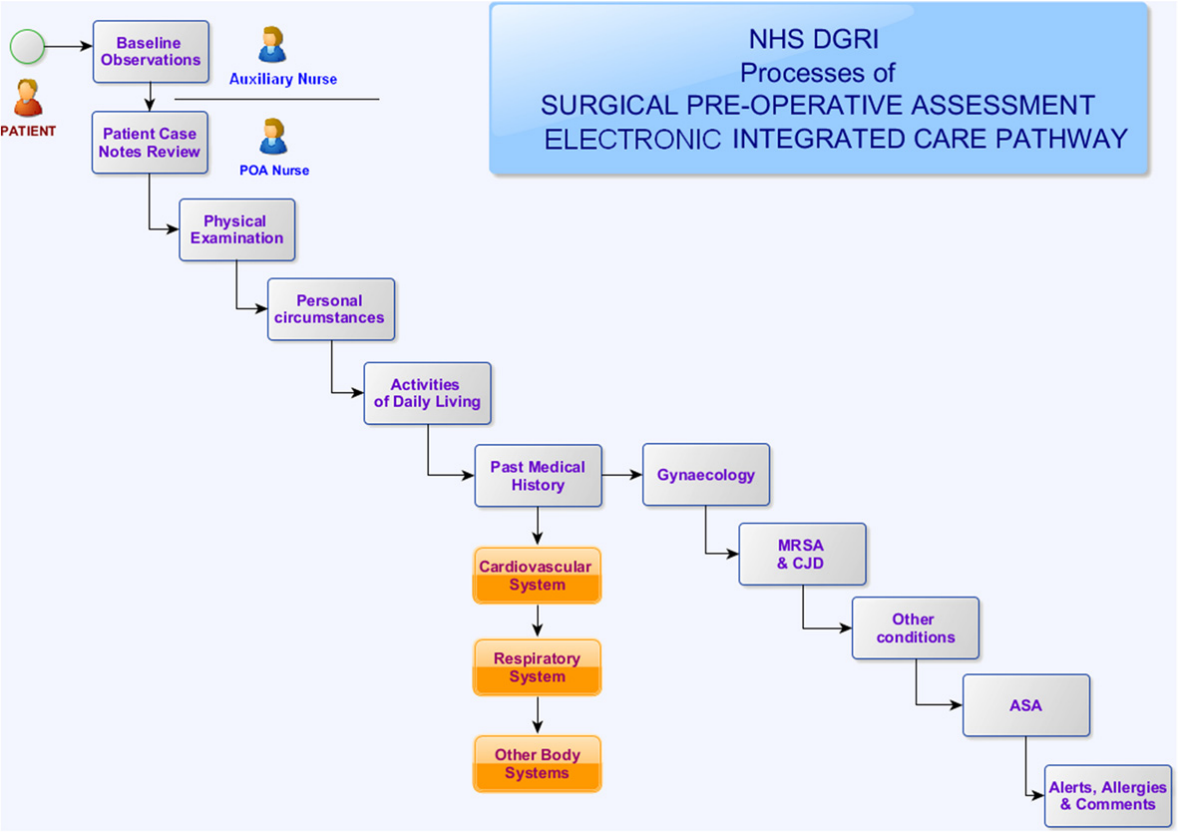
Electronic Preoperative Integrated Care Pathway (Dumfries & Galloway Royal Infirmary - Feb. 2013).

The time allocated for a patient preoperative interview is normally 1 hour. However, it can be as little as 30 minutes for fit, straight-forward patients. The nurse will proceed systematically through the ICP (Figure 3), reviewing in turn: *the patient’s demographic details, hospital management administrative data, baseline patient information, physical examination, including: oral assessment and airway assessment, assessment of personal circumstances and social support, activities of daily living, past medical history, including a review of the main body systems: cardiovascular system, respiratory system, other systems, gynaecology, risk assessment for methicillin-resistant staphylococcus aureus (MRSA) and Creutzfeldt-Jakob disease, other conditions, ASA (American Society of Anesthesiologists) grade, alerts, allergies* & *comments*. 

• *Common morbidities:*

• Nurse 1 suggested that a significant proportion of the population of the Dumfries and Galloway health board are elderly pensioners, from a wide range of socio-economic backgrounds. As a consequence, many patients coming to the clinic were elderly patients with one or more chronic conditions or morbidities (e.g. hypertension, cardiovascular diseases, respiratory diseases and cancer).

• *Current medication:*

• Patients are asked to bring a list of their current medication with them at their appointment. The nurses then type in the list of medication into the ICP. As it is not uncommon for patients to be on multiple medications, this can be a tedious and time-consuming task, as well as being a potential source of transcription errors. During our initial visit, Nurse 1 suggested that a substantial process improvement could be gained if the nurses had direct access to the list of medication from the patient electronic Emergency Care Summary (ECS). The ECS is an electronic patient record summary for unscheduled care. It pulls essential information (medication, allergies and main past medical history) from primary care records and is updated on a daily basis [39]. However, the nurses were not permitted to access the ECS at the time of our initial visit due to the information governance of the ECS, as this record was designed to be accessed only in events of emergency care. However, during subsequent visits, we were informed that the nurses were later provided with the permission to access the patients’ ECS following a special request from the anaesthetic department to the health-board medical director. A permission to access the ECS during routine POA was then formally extended to all the medical and nursing staff in the PAC.

• *Preoperative investigations:*

• The nurses use a range of locally-developed guidelines for deciding on the appropriateness of screening tests, which are in part local implementation of the National Institute for Clinical Excellence (NICE) preoperative guidelines [40]. “Local implementation" means that the guidelines have been implemented after a collegial review by the hospital anaesthetic department and hence, the local protocols may vary slightly from an exact implementation of the original NICE guidelines.

• Ideally, an MRSA screening test is performed during the initial outpatient appointment so that the result is available when the patient subsequently attends the PAC. If the result is positive, then the nurse can provide an eradication treatment immediately. However, the nurses reported that this was not consistently the case and that on occasions, outpatient staff did not perform an MRSA screening test. Patients then had to be screened at the PAC which could introduce further delays. If patients are subsequently tested positive for MRSA, then the patients would need to receive an eradication treatment, either through another visit to the PAC or administered by their primary care doctor.

### Onward referrals from the PAC

Patients can be referred by the nurses or the anaesthetist consultant for further investigations or interventions: 

• Cardiology: The consultant anaesthetist would take the decision for a patient’s referral to cardiology if necessary. The cardiology service was perceived by the PAC staff as being extremely busy and a further referral to cardiology could lead to additional delays before surgery.

• Diabetes clinic: The nurses at the PAC have liaised with the hospital diabetes clinic to develop a pathway to refer diabetic patients for fitness optimisation prior to surgery. The diabetes clinic has contingency bookings for emergency referrals so that a patient can be seen relatively rapidly (e.g. within 6 weeks) for preoperative glycemic control. In the past, diabetic patients were generally admitted as inpatients and put on an insulin sliding scale. At present, the nurses suggested that the preferred option was to admit diabetic patients fasted on the day of surgery.

• Smoking cessation: The nurses routinely encounter smokers with respiratory problems and there is a direct referral pathway from the POA clinic to the smoking cessation service.

### Communication within and outwith the PAC

**Figure 4:**
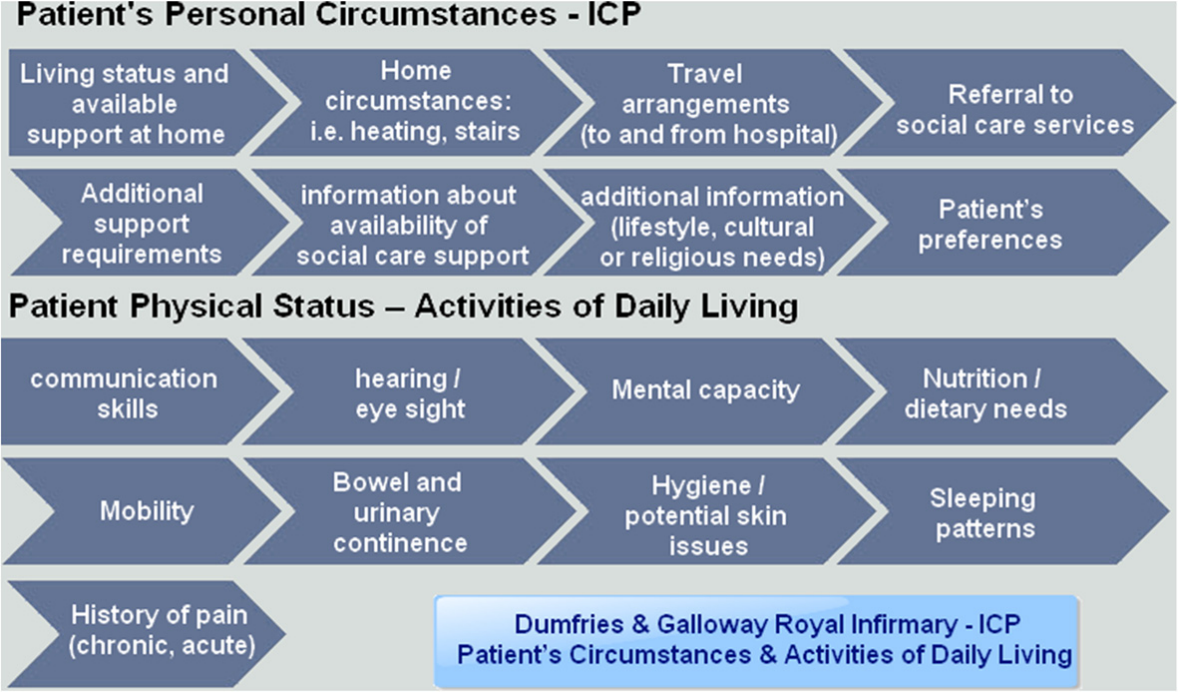
Patient’s circumstances & activities of daily living ((Dumfries & Galloway Royal Infirmary - Feb. 2013)).

• **Communication within the POA multi-disciplinary team:**

• The nurses felt that the anaesthetic department strongly supported the nursing staff, and valued both their experiences and recommendations for individual patient case-management. The nurses also felt that they were able to speak directly and openly with the consultants, as well as raise any clinical concerns if and when appropriate. The auxiliary nurses similarly reported enjoying excellent communication with other members of staff in the service.

• **Nurse 1:***“...because we have an anaesthetist here 3 days a week, we just case-conference with the anaesthetist. [...] If the ECG was abnormal, we could compare with previous ECGs, look at their cardiovascular history, and it would be a discussion with the consultant anaesthetist... who work really closely with the nurses. [...] they basically go around every nurse and see if there’s anything that, if there’s any issues with... patients that we need to discuss with them... and have a one-to-one with each nurse to try and come up with a plan of how we would, you know... overcome issues or indeed if the anaesthetist said 'no that’s fine I am happy with that’, the nurses would then act on any instructions from the anaesthetist. [...] they take on board what the nurses are saying. They realise the nurses are experienced in assessing patients and listen to the nursing staff. Yeah. But equally the nurses don’t feel intimidated about going to speak to an anaesthetist”.*

• **Communication with patients:**

• Caring and patient centeredness are essential elements of the patient-nurse relationship in the perioperative pathway [41]. The patient integrated care pathway was designed from the very outset to be patient-centred (Figure 4). Two important categories of information collected by the nurses during the patient interview include: (i) information on patients’ personal circumstances and a comprehensive section dedicated to (ii) assessing activities of daily living. The routine provision of verbal and/or written information to patients during the POA interview is also explicitly integrated in the patient pathway and documented in the ICP. All patients are also provided with a pre-assessment information leaflet prior to – or during – their interview.

• The standard patient interview duration slot was set to an hour long by the nursing staff to ensure that adequate time was available to collect relevant clinical data but also to provide sufficient time to explain the procedure and respond to questions raised by patients in order to alleviate potential anxieties about the surgery. Nurse 1 considered patient psychological preparation for surgery as an integral part of pre-assessment. This opinion was echoed by the auxiliary nurses who felt that many of the patients could initially be anxious about their operations. However, the POA appointment provided some reassurance and patients valued the opportunity to ask questions and raise concerns during the visit.

• **∙ Nurse 1:***“... they (nurses) need to then have a chat... to the patient about anything, you know, about the surgery. Any questions that the patient may have. Particularly social issues, I think. People sometimes think... don’t realise the implications... they may be off work after surgery or... how they are going to feel post-op? Sometimes they haven’t had much of a chance em... sometimes they haven’t had the chance to do that with the surgeon [...] from a patient’s perspective you know... They want to... sit down and have a chat with the nurse whereabouts it’s relaxed and where they don’t feel intimidated and, you know, the psychological preparation for surgery is a huge part of the nurse’s job in here. And it doesn’t matter if someone’s coming in for a very minor op or major, you know, vascular surgery. Sometimes their anxieties are just the same”.*

• **∙ Aux. Nurse 2:***“... They’re (the patients) given a lot of information before they arrive [...] And we get a lot of feedback from them, don’t they?...saying 'I am glad I came here’, because they’re not as nervous about their operation when they know all the details that are concerned. [...] all the time they’re here, it should be explaining what’s being done to them. Allaying any fears that they have and doing the tests as quickly and efficiently as we possibly can. [...] You’ve got to make it as smooth and easy for the patients as you possibly can”.*

• A review of the literature also emphasised the importance of nurse-patient communication to effectively manage patients’ potential anxieties around surgery and post-operative recovery [42]. Several studies have suggested positive associations between patient preoperative education and postoperative outcomes [43-46]. Patients value the opportunity to be provided with personalised information which provides some psychological preparation and helps them cope with the experience of the surgical procedure [47-50]. Rosen et al. suggested that patients experiencing discomfort following day-case surgery considered insufficient information as one the potential factors affecting their perceptions of post-operative recovery [51].

• **Communication with primary care:**

• The nurses suggested that communication with GPs was not always straightforward. It was not unusual for them to have to follow-up previous correspondence on treatment requests with phone-calls, even when they had specifically requested explicit feed-back on treatment progress so that patients could be cleared for surgery. Occasionally, the GPs responded directly to the surgical consultant and if the consultant then did not relay that information back to the PAC, then the patient could find themselves in an administrative 'limbo’. They could not be cleared for surgery by the nurses until they had received confirmation of the patient’s treatment. Surgery could then be substantially delayed, not because of the patient’s health status, but for the lack of adequate communication and information sharing between the various services involved. The nurse therefore suggested that being able to communicate with GPs in a more formal way, using direct electronic communication would facilitate and speed-up their processes.

### Information management processes in the PAC

• **Development of an electronic integrated care pathway:**

• Prior to the opening of the PAC, the possibility of purchasing a commercial information software for managing the preoperative documentation was investigated but no suitable product was identified at the time. A decision was therefore made by the PAC working group to initiate the development of an in-house electronic system with the support of the health-board NHS IT staff. A key motivation behind the application development was to design a system which would meet the information needs of the various members of the MDT and establish paperless processes within the PAC. Members of the POA MDT worked closely with the IT team to develop the system by providing specifications for clinical content as well as functional and usability requirements. The POA system specifications are continuously updated by the PAC staff, which are then communicated to the IT team and iteratively implemented.

• Initially, some of the nurses were slightly apprehensive of using a computer system during the patient interview due to a lack of familiarity with computers as well as concerns about the potential impact on the patient’s experience and satisfaction during the appointment. However, within a relatively short time, the nurses were quite comfortable using the system and the computer was not deemed detrimental to the patients’ experiences. Additional substantial benefits of the system include the standardisation of processes and the production of legible clinical documents which facilitated information access and sharing across the MDT.

• **Nurse 1:***“[...] we work really closely with IT and they... we inform what’s needed to go into the pre-assessment database and they set it all up for us. So it’s really good. Nurses, you know, who aren’t used to working with computers... and six months after we opened, the system was down to get re... to get upgraded. Every one of the nurses said: 'please can we have the computers back!’. So... They thought it would be quite difficult having a relationship with the patient whilst typing rather than talking to the patient. Actually: it is fine! It is no different. Paper work is legible [...] but yes the system is really good... very good”.*

• **Issues around integration of information management systems:**

• Several electronic information systems co-exist in parallel within the hospital but these are not currently integrated: 

 - a patient management system is used to book POA appointment and list patients for surgery (TOPAS, The Open Patient Administration System)

 - a POA electronic information management system (in-house web-based software development)

 - an electronic laboratory system is available to access screening test results

 - a surgical theatre management system.

• The nurse suggested that it would be very useful to have all the systems fully integrated. In her opinion, this would eventually be implemented once a new electronic patient record system was finally introduced in the hospital [39]. Although the POA documentation is essentially paper-less within the PAC, the clinical documents ultimately need to be printed out to be sent out to the surgical wards. One concern was the size of the assessment documentation once printed. Anaesthetist 1 was of the opinion that if the POA document could be summed up in a succinct summary of key findings, this could considerably improve information-sharing with the surgical wards as well as streamlining the patients’ case-notes reviews by anaesthetists.

• **∙ Anaesthetist 1:***“[...] I think one of the big issues now is... because of the medico-legal... even if you’re fit and well, you still have to answer lots and lots of questions about... even though they’re all things we know... about risks of pressure-sores, any nutritional problems... so even though you know... a 25 year old guy with no problems at all... This is the kind of paperwork you know... this is for everybody. This is for everyone that comes needs, to have it done. A 20 year old man that... you know: 'how are you going to get home?’... and we’re in danger of drowning in paper work you know”.*

These concerns will be in part addressed by the PAC migrating to new information management systems (i.e. the TrakCare patient administration system and clinical portal) supplied as part of the roll-out of the Scotland eHealth programme [39]. These new systems will be able to provide comprehensive access to the patients’ clinical documents in a variety of electronic repositories.

### Inpatient vs. day case elective BADS procedures in the NHS Dumfries & Galloway health-board

The Dumfries & Galloway health-board patient population was estimated to be approximately 150,828 as of June 2012 [52]. The British Association of Day Surgery (BADS) has developed a Directory of Procedures which now includes over 200 recommended day and short stay surgical procedures, coded and categorised by surgical specialty [53].

Table 1 present the number and ratio of inpatient vs. day case elective BADS procedures in the NHS Dumfries & Galloway for the 5 financial years 2008/09 to 2012/13. The figures were obtained from the Operations and Procedures - Hospital Care data set published in September 2013 by the Information Services Division, ISD Scotland, the statistical services of NHS Scotland.

**Table 1:** Elective BADS procedures, in-patient, outpatient and day-case activity for 2008/09 to 2012/13 in NHS Dumfries & Galloway Health-Board

**Indicator**	**2008/09**	**2009/10**	**2010/11**	**2011/12**	**2012/13**
Total elective BADS procedures	8,251	8,213	8,959	8,218	7,967
BADS procedures as inpatients	1,677	1,600	1,472	1,454	1,245
BADS procedures as day cases	5,936	6,236	5,586	5,257	5,023
BADS procedures as outpatients	638	377	1,901	1,507	1,699
Percentage BADS procedures	79.7%	80.5%	83.6%	82.3%	84.4%

[source Information Service Division, ISD, Operations and Procedures - Hospital Care, Sept. 2013. Available at: http://www.isdscotland.org/Health-Topics/Hospital-Care/Operations-and-Procedures/. File: Annual_trends_in_BADS_procedures_hbt_Sep13.xls (last checked in March 2014)].

The procedure activity trend presented applies to the whole health-board and not exclusively to DGRI. However, DGRI is the main acute care hospital for the region, and the PAC is the main preoperative clinic in the health-board, with a second smaller service also provided in Stranrear hospital. Thus, the figures present a useful proxy measure of day-case procedures activity in the health-board since the DGRI PAC opened in July 2008.

## Interpretation & discussion

Using the 4 NPT constructs, we review and interpret the findings of this study in turn:

### Coherence

Coherence refers to the “sense-making” work undertaken when a new health intervention is implemented: to determine whether users see it as differing from existing practice, have a shared view of its purpose, understand how it will affect them personally and grasp its potential benefits [54]. Coherence with regards new service implementation include policy building or dissemination of information, undertaken either locally or nationally.

The rationale for preoperative services redesign in DGRI was well established due to the recurrent problems associated with the traditional inpatient route without pre-assessment, such as insufficient patient preparation or late surgery cancellations. In addition, the PAC development was supported by national health-policy initiatives combined with a range of performance targets (HEAT targets for RTT and BADS surgery procedures). In 2006, the Planned Care Improvement Programme (PCIP) aimed to improve the flow of patients along their healthcare pathways through sustainable clinical systems improvement [20]. The PCIP set 'active admission management’ as one of five key strategic priorities. This was a key driver for the development and streamlining of PACs across NHS Scotland. NHS Borders, Dumfries & Galloway, Orkney, Shetland and Tayside developed new PACs while NHS Greater Glasgow and Clyde, Highland, Lanarkshire, Lothian, Tayside and Western Isles undertook to streamline and standardise pre-assessment procedures and services [55].

Coherence within the PAC is high but is less so at the interface with other services within the health-board. The nurses felt that the service was patient-centred and considered a thorough assessment and patient information and education as central to POA. The anaesthetist considered POA to be a filtering process: to detect early-on potential problems in order to effectively manage the planed admission route for surgery. This is a key step to assess whether the patient can be admitted for day-case surgery or 23-hours care. In that respect, the anaesthetist perceived a lack of overall coherence for assessment depending on the various hospital admission routes. Junior doctors were not routinely involved or indeed present at the PAC during pre-assessment and clerking of patients but were however involved in specific patient admission routes (i.e. 23-hours care patients for non-orthopaedic surgery and inpatients). The anaesthetist considered that the various assessment pathways, depending on the type of patient admission, were confusing for the POA staff. His opinion was that junior doctors did not need to be involved at all during patients’ assessment and that the involvement of nurses, supported by the weekly anaesthetic clinics, was entirely sufficient for the effective assessment of all patients.

In addition, the anaesthetist considered that the scope to increase the number of day-surgery admissions as DGRI remained substantial. He also suggested that some patients who were deemed suitable for day-surgery or 23-hours care at the PAC were still admitted through the inpatient route by surgeons. This, in his view, was not justified by clinical reasons but possibly motivated by logistic reasons, such as hospital bed-management policies. This concern was also raised at several other PACs visited during this study. The most likely explanation is that an admission as a day-surgery patient does not guarantee a bed assignment following surgery, which could then introduce further delays in recovery theatres. Interestingly, this potential drawback in day-case patient care management has also recently been identified in a separate study of a surgery admission unit [56].

### Cognitive participation

Cognitive participation focuses upon the work undertaken to engage with potential users and get them to “buy into” a new intervention [54]. Clinical pathways redesign, focused on patient assessment, improved communication within the MDT, improved planning and management, and patient participation have shown the potential to be effective interventions in reducing the number of surgical cancellations [57,58]. However, it is also essential that clinical staff understand the rationale for changes, and are actively engaged in service redesign, as previous studies have suggested that frequent reorganisation of services can lead to *'change fatigue’*, staff disaffection and poor morale [59,60].

The PAC development was steered by a multi-disciplinary team, spanning a range of hospital departments. The clinic design itself was nurse-led, with considerable support from the anaesthetic department and hospital management. The PAC integrated highly experienced nursing staff from the hospital day-surgery unit. From the outset, the hospital IT staff worked closely with the PAC staff to develop an in-house electronic ICP which met local needs and priorities. The opening of the PAC using a computerised system substantially transformed clinical practices in pre-assessment and following a teething period of approximately a year, the nursing staff were entirely satisfied with the running and work practices at the clinic. Involvement of frontline clinical staff in process-redesign across specialties and the use of computer application to improve management of patient and care planning has been shown to promote successful surgical pathways redesign interventions [37,58].

### Collective action

The emphasis of collective action involves the work performed by individuals, groups of professionals or organisations in operationalising a new intervention in practice and socio-technical issues, such as how new systems affect the everyday work of individuals, organizational structures and goals [54]. Clinical care pathways are often local implementations of standardised regional and national guidelines in response to contextualised priorities. A successful clinical pathway implementation requires that all the individuals involved in the setting up of the new service have an opportunity to define their own roles in terms of *responsibilities and relationship* to others, fostering both a sense of participation and accountability [37].

The nursing staff were already highly experienced in the care and management of day-case surgical patients and received additional contextualised POA competency training before joining the new PAC. Roles and responsibilities are clearly defined and communication across the members of the MDT appeared excellent, with nurses feeling confident and able to communicate any concerns to consultants during the weekly anaesthetist-led clinics. This aspect of the PAC is essential as a previous review has suggested that effective communication and information sharing across the perioperative pathway is essential for the delivery of safe outcomes for surgical patients [5].

At the organisation-wide level, the anaesthetist suggested that a more standardised approach to data collection during the pre-assessment of patients across NHS hospitals would bring much needed guidance and clarity to the service and facilitate the work of the PAC. In addition, a lot of the information collated at the PAC is already available in primary care. Therefore, he further suggested that there was some scope for improvement if, for example, some of the pre-assessment tasks were carried out in primary care. While some of the anaesthetist’s concerns regarding information access could be in principle addressed through improved integrated information systems – the example of the PAC staff being granted permission to access the ECS during the routine assessment of patients being such an example – the matter of clearer national guidance for POA can only be effectively addressed through the development of national guidelines or policy programmes.

### Reflexive monitoring

Reflexive monitoring deals with the evaluation and monitoring of health interventions and how these are used to influence utilisation in future [54].

There was no formal continuing professional development and education system in place at the PAC and much of the knowledge transfer between staff took place in the course of their duties. The weekly anaesthetist-led clinics were perceived as an important forum for this. Although the clinical staff would have liked to set some time aside for regular continuing professional development sessions, work-load pressures and the need to meet a range of targets (particularly RTT) made this unlikely at a time when the NHS is coming under considerable resource constraints.

There was no formal assessment of the effectiveness of the service in terms of impacts on cancellation rates or perioperative complications. The PAC staff will only receive informal feed-back from the surgical wards when there is an unexpected late operating theatre (OT) cancellation. However, it is not currently possible for the PAC staff to identify the reasons for late cancellations as this information is not adequately coded in the OT information management system. This prevents them from distinguishing patients whose surgery is cancelled due to an unpredictable medical event (e.g. infection not present at the time of POA), due to hospital resources management, or a genuine health-related issue which ought to have been detected during the patient assessment and was somehow missed.

∙ **Anaesthetist 1:***“[...] what I want to see now is... how many patients are being cancelled on the day of surgery?...and so... what I get every week is a list of patients, but unfortunately because of the software in the theatre management... is very blunt. All it says is: 'patient not fit’....and up to now we haven’t been able to improve the system, so in other words: I get a list of reasons, but they may not be fit because on the day of surgery they may have a bad cold which is fair enough. But what I want to know is: 'are they cancelled because they’ve had inadequate pre-operative assessment?"*

The clinical staff hoped to see an improved perioperative monitoring system being set up at the hospital in the near future. They considered that this could contribute to the improvement of POA by providing formal feed-back on the PAC performance, such as monthly cancellation rates with a clear explanation associated with each cancellation. They also suggested that the imminent introduction of a new patient administration system and improved coding in the surgical wards would allow for such an auditing system to be developed in due course.

### Conclusion

Interpreting our results through the lens of NPT is useful in understanding the combination of factors which led to the successful development of an integrated PAC in the Dumfries & Galloway Royal Infirmary. An important motivation for the development of the PAC was to provide a more coherent pathway for patient assessment for surgery across the hospital and health-board, to reduce late surgical cancellations, increase the ratio of day-case and outpatient surgery and improve the overall preoperative assessment of patients. Service redesign was not only considered a clinical priority by members of the anaesthetic department but also as an operational priority by members of the hospital management, which is evidenced by the resources and preparation which were allocated for the design and development of the PAC. The PCIP provided both the strategic impetus and some resources to improve the patient journey into secondary care. In addition, the British Association of Day Surgery procedures and the Referral-To-Treatment HEAT targets meant that all NHS boards were bound to operationalise improvements to the patient surgical pathway as part of their Local Delivery Plans in order to meet these targets. It was thus the synergies of both local priorities – as exemplified by the agency of stakeholders on the ground – with national strategic priorities which have enabled the successful deployment and normalisation of innovative clinical and information management processes in the DGRI PAC. The development of a new electronic information management system was integral to the design of the new PAC. The system was developed collaboratively by the POA staff and the health-board IT team, resulting in a highly contextualised operationalisation of clinical and information management processes in the PAC. The POA staff have reported process-related benefits as the result of having an integrated PAC and electronic information system and ISD BADS procedures figures also show an increase in the ratio of day-case procedures performed in the health-board since the opening of the PAC.

The main limitation of our study is that we were not able to collect data on pre-assessment or surgical delays, cancellations or outcomes. Hence, the impact of the PAC on clinical outcomes is unclear. Therefore, a substantial – yet unfulfilled – potential benefit in embedding the use of information technology in routine use within preoperative clinics would be to improve the overall reporting of surgical outcomes.

## Abbreviations

ASA: American society of anesthesiologists (grade); BADS: British association of day surgery; DGRI: Dumfries & Galloway royal infirmary; ECS: Emergency care summary; GP: General practitioner; HEAT: Health, Efficiency, Access and Treatment (targets); ICP: Integrated Care Pathway; ICT/IT: Information & Communication technology; ISD Scotland: Information services division, the statistical services of NHS Scotland; MDT: Multi-disciplinary team; MRSA: Methicillin-resistant staphylococcus aureus; NCEPOD: National Confidential Enquiry into Patient Outcome and Death; NICE: National institute for clinical excellence; NHS Scotland: National health service for Scotland; NPT: Normalisation process theory; OT: Operating theatre; PAC: Pre-operative assessment clinic; POA: Pre-operative assessment; RTT: Referral-to-treatment (18 weeks target); TOPAS: The open patient administration system.

## Competing interests

The authors declare that there are no conflicts of interest.

## Authors’ contributions

M-MB and FSM conceptualized the project. M-MB conducted all visits on the site and conducted the semi-structured interviews, data collection and analysis, performed the literature and internet searches and drafted the original submission of this manuscript. FSM critically reviewed and revised subsequent versions of the manuscript. Both authors read and approved the final manuscript.

## Pre-publication history

The pre-publication history for this paper can be accessed here:

http://www.biomedcentral.com/1472-6947/14/22/prepub
